# Long noncoding RNA H19 regulates the therapeutic efficacy of mesenchymal stem cells in rats with severe acute pancreatitis by sponging miR-138-5p and miR-141-3p

**DOI:** 10.1186/s13287-020-01940-z

**Published:** 2020-09-25

**Authors:** Guodong Song, Jia Zhou, Ruimei Song, Dalu Liu, Weidi Yu, Wangcheng Xie, Zhilong Ma, Jian Gong, Hongbo Meng, Tingsong Yang, Zhenshun Song

**Affiliations:** 1 Department of General Surgery, Shanghai Tenth People’s Hospital Affiliated to Tongji University School of Medicine, Shanghai, 200072 China; 2 Tongren Hospital, Shanghai Jiao Tong University School of Medicine, Shanghai, 200336 China

**Keywords:** Long noncoding RNA H19, Mesenchymal stem cells, Severe acute pancreatitis, Autophagy, Cell proliferation

## Abstract

**Background:**

Patients with severe acute pancreatitis (SAP), which is characterized by high morbidity and mortality, account for an increasing medical burden worldwide. We previously found that mesenchymal stem cells (MSCs) could attenuate SAP and that expression of long noncoding RNA H19 (LncRNA H19) was upregulated in rats receiving MSCs. In the present study, we investigated the mechanisms of LncRNA H19 regulating the therapeutic efficacy of MSCs in the alleviation of SAP.

**Methods:**

MSCs transfected with LncRNA H19 overexpression and knockdown plasmids were intravenously injected into rats 12 h after sodium taurocholate (NaT) administration to induce SAP.

**Results:**

Overexpressing LncRNA H19 in MSCs significantly enhanced the anti-inflammatory capacity of the MSCs, inhibited autophagy via promotion of focal adhesion kinase (FAK)-associated pathways, and facilitated cell proliferation by increasing the level of β-catenin in rats with SAP. LncRNA H19 functioned as a competing endogenous RNA by sponging miR-138-5p and miR-141-3p. Knocking down miR-138-5p in MSCs increased the expression of protein tyrosine kinase 2 (PTK2, encoding FAK) to suppress autophagy, while downregulating miR-141-3p enhanced the level of β-catenin to promote cell proliferation.

**Conclusions:**

In conclusion, LncRNA H19 effectively increased the therapeutic efficacy of MSCs in rats with SAP via the miR-138-5p/PTK2/FAK and miR-141-3p/β-catenin pathways.

## Introduction

Acute pancreatitis (AP) is an inflammatory disease of the pancreas that causes considerable morbidity and mortality [1]. The global incidence of AP is approximately 30 cases per 100,000 persons and has been increasing universally [2]. In addition, about 20% of AP cases evolve into severe acute pancreatitis (SAP), with a mortality rate of 15% [3]. The incidence of SAP and its complications has rapidly increased [4].

While the underlying mechanisms of SAP are still not completely known, there is some consensus regarding its initiation. SAP begins with the activation of digestive enzymes in pancreatic acinar cells leading to cell injury [5]. It has been reported that systemic inflammatory responses and anti-inflammatory responses develop in parallel during the progression of SAP [6]. Some of the proinflammatory cytokines involved in the inflammatory responses of SAP are interleukin-1β (IL-1β), IL-6, IL-8, and tumor necrosis factor alpha (TNF-α) [7, 8]. In recent years, increasing evidence has demonstrated that impaired autophagy has a crucial effect on the pathogenesis of SAP [9–11]. In addition, maintaining the normal action of adhesion molecules (such as β-catenin) and restricting their abnormal activation are beneficial in blocking the development of AP [12]. Therefore, investigations seeking to develop novel therapeutic tactics for SAP should concentrate on how to inhibit inflammatory responses, regulate autophagy, and maintain levels of adhesion molecules.

Treatment of patients with SAP requires synergistic action from various hospital departments, including gastroenterology, surgery, and critical care medicine [4]. Unfortunately, these treatments are often invasive and result in more complications. Hence, a noninvasive and effective therapy is required, and we suggest that mesenchymal stem cells (MSCs) be considered as a treatment option. MSCs have been applied in a variety of difficult diseases including musculoskeletal tissue injuries [13], myocardial infarction [14], and Crohn’s disease [15]. Jung et al. previously confirmed that human bone marrow-derived clonal MSCs could suppress inflammatory responses in rats with AP [16]. Our team has elucidated some of the important mechanisms by which MSCs attenuate SAP [17–24]. We found that MSCs can ameliorate SAP-associated multiple organ injury via suppression of autophagy [25], and we also detected statistically significant differences in the expression of long noncoding RNA H19 **(**LncRNA H19). LncRNAs > 200 bp in length are involved in many biological processes, and in fact, LncRNA H19 is an imprinting lncRNA which can facilitate tamoxifen resistance in breast cancer by upregulating autophagy [26]. LncRNA H19 has been confirmed to increase the therapeutic efficacy of MSC-derived exosomes in acute myocardial infarction [27], and the result provided us with significant hints in MSCs treating SAP. Based on our previous findings, we hypothesized that LncRNA H19 may also play an important role in SAP. The current study was carried out to clarify the possible mechanisms by which LncRNA H19 may regulate the therapeutic efficacy of MSCs attenuating SAP.

## Materials and methods

### Cell culture

Four-week-old SD rats were sacrificed, and the primary MSCs were isolated from the femurs and tibias under a sterile condition. Following flushing the bone marrow cavity with DMEM-LG medium (Middleton, WI, USA) and removing large tissues with a 200-mesh nylon filter, the isolated bone marrow cells were cultured in DMEM-LG medium supplemented with 10% fetal bovine serum (Gibco, NY, USA) and 1% penicillin (Gibco) at 37 °C in an environment with 5% CO_2_. The medium was refreshed every 3 days, and cells were harvested by multiple digestions and passaged when cell confluence reached > 80%. Cells of the fifth passage were collected and subjected to flow cytometry analysis to identify. 293T cells were obtained from the Chinese Academy of Sciences (Shanghai, China) and seeded in DMEM media (Gibco) with 10% fetal bovine serum (Gibco). All cells were incubated in a humidified atmosphere at 37 °C with 5% CO_2_.

### Plasmid, siRNA, and transfection

The overexpression full-length plasmid of LncRNA H19 (pcDNA-H19) was acquired by inserting LncRNA H19 into the pcDNA3.1 vector (Invitrogen, Shanghai, China). Small interfering RNA (siRNA) targeting rat LncRNA H19 (si-H19), miRNA negative control (miR-NC), miRNA mimics, and inhibitors for rno-miR-138-5p and rno-miR-141-3p were purchased from GenePharma (Shanghai, China). Short hairpin RNAs (shRNA) of PTK2 (sh-PTK2) and β-catenin (sh-β-catenin) were constructed using pGPU6 plasmids (GenePharma). PTK2 and β-catenin overexpression plasmids were constructed with pEX2 plasmids (GenePharma). siRNA, miR-NC, miRNA mimics, and inhibitor were transfected into cells using HiPerFect Transfection Reagent (QIAGEN, Valencia, CA, USA) according to the manufacturer’s instructions. Plasmids were transfected into cells by using Lipofectamine 3000 (Invitrogen) following the manufacturer’s protocol.

### In vivo protocol

Healthy male Sprague-Dawley rats (4–6 weeks old) were obtained from Shanghai Laboratory Animal Co., Ltd. (Shanghai, China). All animal experiments complied with the National Institutes of Health Guide for the Care and Use of Laboratory Animals (NIH Publications No. 8023, revised 1978). All experimental protocols were approved by the Institutional Animal Ethics Committee of the Shanghai Tenth People’s Hospital, affiliated to Tongji University School of Medicine. The 30 rats were randomly divided into five groups (*n* = 6 per group) for animal experiments as follows: (1) negative control (NC) group; (2) SAP group—animals were administered sodium taurocholate (NaT) as previously described [23]; (3) MSC group—MSCs (1 × 10^7^ cells/kg body weight) suspended in 300 μL of phosphate-buffered saline (PBS) were transplanted into SAP model rats via the tail vein 12 h after treatment with NaT; (4) pcDNA-H19-MSC group—pcDNA-H19-MSCs (1 × 10^7^ cells/kg body weight) were injected into SAP rats as described for the MSC group; and (5) si-H19-MSC group—si-H19-MSCs were treated as described above. In addition, different MSC-treated groups (*n* = 6 per group) were administered various MSCs that had been transfected with miR-NC, miRNA mimics, miRNA inhibitor, or expression plasmids. All animals were euthanized 72 h after induction of SAP. They were euthanized by intraperitoneal injection of 10% pentobarbital (1 ml/kg body weight). A supplementary image with the experimental groups would help the reader to follow the workgroups (Additional file 1).

### Histopathologic examination

After euthanization, fresh rat pancreatic tissues were excised and preserved in 4% paraformaldehyde. Tissues were embedded in paraffin and stained with H&E for histological evaluation. The severity of pancreatic injuries was scored by two experienced and blinded pathologists as previously described [23].

### Biochemical inspection of serum and tissues

The levels of serum amylase activity were analyzed using colorimetric assay kits (BioVision, Milpitas, CA, USA), according to the manufacturer’s instructions. The levels of mediators in serum and pancreatic tissues (LDH, IL-1β, IL-6, IL-8, and TNF-α) were detected using ELISA kits (R&D Systems, Minneapolis, MN, USA) following the manufacturer’s protocol.

### Flow cytometry

The survival rate of MSCs was detected using a flow cytometry assay, as previously described [23]. Briefly, collected MSCs were washed twice with PBS and incubated with FITC-Annexin V (BD Pharmingen, San Diego, CA, USA) and propidium iodide (BD Pharmingen). The percentages of live MSCs were determined by flow cytometry (BD Biosciences, San Jose, CA, USA). The Q4 quadrant represents live MSCs.

### Quantitative real-time polymerase chain reaction

Total RNA was extracted from frozen pancreatic tissue by means of TRIzol reagent (Invitrogen). According to the instructions of the PrimeScript Reverse Transcriptase Reagent Kit (Kapa Biosystems, Boston, MA, USA), RNA was reverse-transcribed to cDNA. The qRT-PCR assays were conducted using a KAPA qPCR Kit (Kapa Biosystems). GAPDH and U6 were utilized as the endogenous controls. The primer sequences are listed in Table 1. Relative expression of various RNAs and genes was determined using the comparative 2^−ΔΔCT^ method as previously described [23].

**Table 1:** Primer sequences for qRT-PCR

Gene/RNA	Forward (5′-3′)	Reverse (5′-3′)
LncRNA H19	CGTTCCTTTAGTCTCCTGAC	AGTCCGTGTTCCAAGTCC
PTK2	GTGCTCTTGGTTCAAGCTGGAT	ACTTGAGTGAAGTCAGCAAGATGTGT
β-Catenin	TGATAAAGGCAACTGTTGGATTGA	CCGCTGGGTGTCCTGATGT
GAPDH	CGCTAACATCAAATGGGGTG	TTGCTGACAATCTTGAGGGAG
Primers	rno-miR-138 (5′-3′)	U6 (5′-3′)
Forward	AGCUGGUGUUGUGAAUC	GTAGTCGGCGAAGGTCTCAC
Reverse	GTGCAGGGTCCGAGGT	ACCGTGGATGCAATGCCTAA
Primers	rno-miR-141 (5′-3′)	U6 (5′-3′)
Forward	UAACACUGUCUGGUAAAGAUGG	AACGTTCACGAATTTGCGT
Reverse	CAUCUUCCAGUACAGUGUUGGA	CTCGCTTCGGCAGCACA

### Western blotting

Total protein was extracted from tissues and cells using RIPA lysis buffer (Invitrogen) with PMSF (1100, Beyotime, Nantong, Jiangsu, China), as previously described [23]. Equal amounts of proteins were transferred to nitrocellulose membranes, which were imaged on an Odyssey scanner (LI-COR Biosciences, USA) after incubation with primary and secondary antibodies. Primary antibodies, purchased from CST (Danvers, MA, USA), were as follows: focal adhesion kinase (FAK), phosphoinositide-dependent kinase 1 (PDK1), protein kinase B (AKT), phosphorylated AKT (p-AKT), mammalian target of rapamycin (mTOR), phosphorylated mTOR (p-mTOR), P62, Beclin-1, microtubule-associated protein 1 light chain 3 (LC3), β-catenin, c-Myc, and cyclin D1. The dilution of primary antibodies was 1:1000. Secondary antibodies used were anti-rabbit IgG (CST) and anti-mouse IgG (CST). The dilution of secondary antibodies was 1:2000.

### Immunohistochemistry

Immunohistochemistry was conducted as previously described [23]. Tissue sections were incubated with primary antibodies (all purchased from Abcam, Cambridge, UK) as follows: FAK, PDK1, AKT, p-AKT, mTOR, p-mTOR, P62, Beclin-1, LC3, β-catenin, c-Myc, and cyclin D1.

### Immunofluorescence

Immunofluorescence staining was performed on 5-μm pancreatic tissue sections as previously described [25]. Primary antibody LC3 (Abcam) was utilized following the manufacturer’s introductions.

### Transmission electron microscopy

Cells in the pancreas were observed by transmission electron microscopy (JEM 1230, Tokyo, Japan) after preservation in glutaraldehyde buffer and fixation in osmium tetroxide, as previously described [25]. The detailed steps of TEM are as follows: (1) place the samples, (2) adjust magnification, (3) take pictures, (4) photograph diffraction, (5) analyze energy spectrum, and (6) high resolution.

### Dual-luciferase reporter assay

Fragments of the 3′-UTR of LncRNA H19, PTK2, and β-catenin were synthesized with binding sites for rno-miR-138-5p or rno-miR-141-3p containing wild-type or mutant sequence. These fragments were extended by PCR and inserted downstream from the luciferase gene in the psiCHECK-2 vector (Promega, Madison, WI, USA). For luciferase reporter assays, 293T cells were plated in 6-well plates at a density of 5 × 10^5^ cells/well, then co-transfected with rno-miR-138-5p mimics, rno-miR-141-3p mimics or negative control (miR-NC), and luciferase reporter vectors using Lipofectamine 3000. Luciferase activity was measured using a luciferase reporter assay kit (Promega) 48 h after transfection according to the manufacturer’s instructions.

### RNA pull-down assay

Biotinylated probes, which contained reverse complementary sequences to LncRNA H19 black-splice junction sequences (LncRNA H19 probe), were provided by Sangon Biotech (Shanghai, China). MSCs (1 × 10^7^) were washed using cold PBS, lysed in RIP lysis buffer (Invitrogen), and incubated for 2 h at 25 °C with specific RNA probes, labeled with high-affinity biotin. Next, the suspension and streptavidin magnetic beads (Thermo Fisher Scientific, Shanghai, China) were mixed and incubated for 1 h at 25 °C and washed twice with wash buffer. In the end, the RNA was analyzed via agarose gel electrophoresis and quantified using qRT-PCR assays as previously described [28].

### FISH

FISH reveals the abundance and positioning of nucleic acid sequences in cells or tissues [29]. MSC nucleus staining was performed using 4,6-diamidino-2-phenylindole (DAPI, Beyotime, Shanghai, China). Fluorescein isothiocyanate (FITC) probes were specific to LncRNA H19. All procedures were performed following the manufacturer’s protocol (Biofavor, Wuhan, Hubei Province, China).

### Statistical analysis

All data are presented as means ± standard deviation (SD) from at least three independent experiments. Statistical analysis was conducted using one-way analysis of variance (ANOVA) and unpaired Student’s *t* tests. A *p* value < 0.05 was considered statistically significant.

## Results

### LncRNA H19 enhanced the therapeutic efficacy of MSCs on SAP

**Fig. 1:**
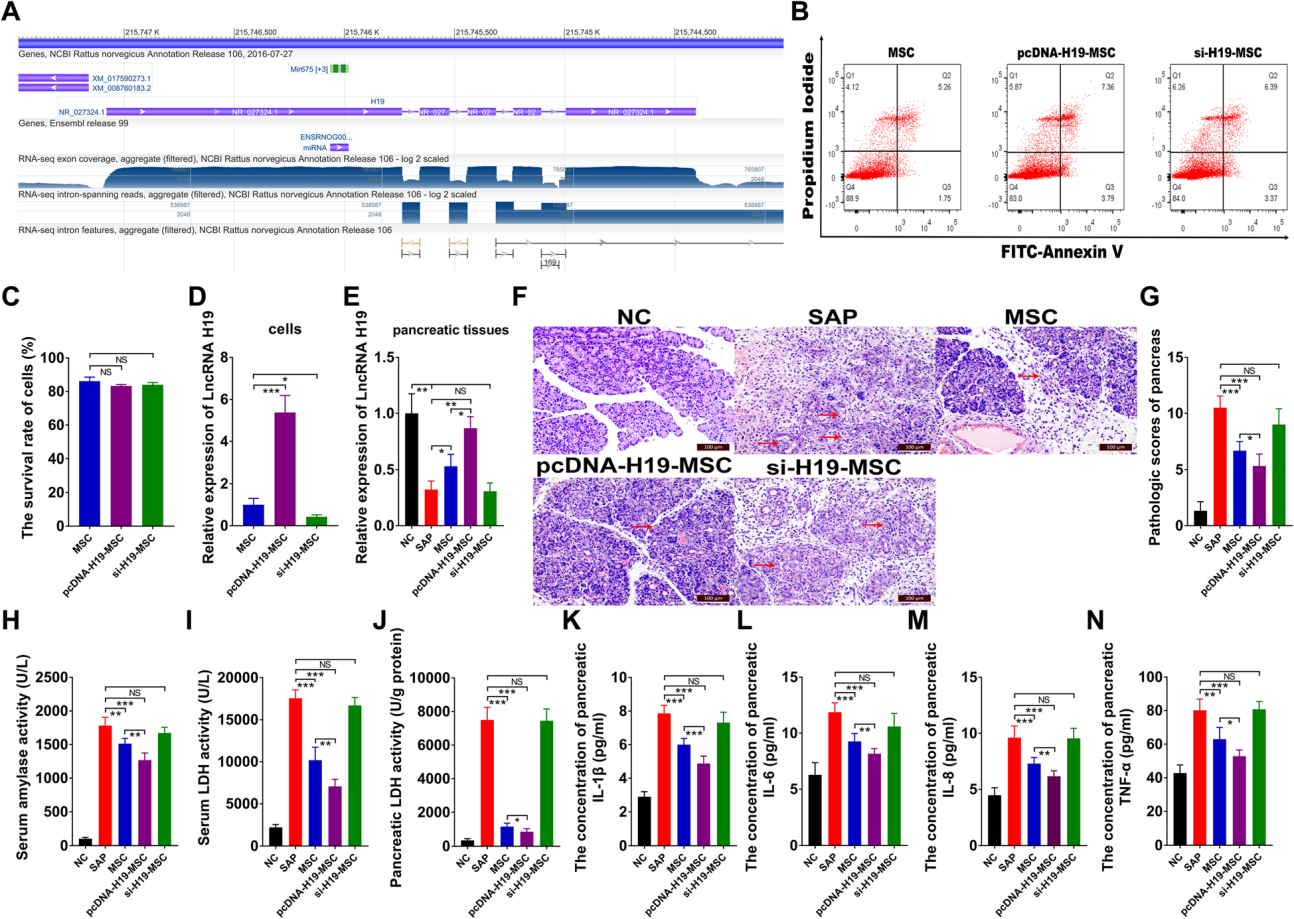
LncRNA H19 upregulated the therapeutic efficacy of MSCs on SAP. **a** Information about genomic regions, transcripts, and products of LnRNA H19 (*Rattus norvegicus*). **b** Allocation of necrotic, apoptotic, and live MSCs, detected by flow cytometry. **c** The survival rate of MSCs determined by flow cytometry. **d**, **e** Relative mRNA expression of LnRNA H19 in MSCs and pancreatic tissues analyzed by qRT-PCR, respectively. **f** H&E staining of pancreatic tissues (scale bar = 100 μm) (red arrowhead indicates necrosis of pancreatic lobules and inflammatory cell infiltration). **g** Pathological scores for H&E staining in the pancreas. **h** Levels of serum lipase activity. **i** Levels of serum LDH activity. **j** Levels of pancreatic LDH activity. **k**–**n** Concentration of IL-1β, IL-6, IL-8, and TNF-α in the pancreas. Data represent means ± SD from at least three independent experiments. *n* = 6/group. *p < 0.05, **p < 0.01, ***p < 0.001. NS, not significant

To investigate the specific lncRNAs involved in the pathological process of SAP, we detected the expression of lncRNAs using quantitative real-time PCR (qRT-PCR) after treating normal rats with NaT to induce SAP. Xia et al. have demonstrated that lncRNAs uc.308-, BC158811, BC166549, BC166474, and BC161988 were significantly suppressed by NaT administration [30], but we also found a novel lncRNA, LncRNA H19, which was prominently inhibited in the SAP group. Detailed information about LncRNA H19 (rat species) can be obtained from the website https://www.ncbi.nlm.nih.gov/gene/309122 (Fig. 1a). We determined the survival rate of MSCs under different treatments using flow cytometry and found that overexpressing or knocking down LncRNA H19 did not affect the survival rate of MSCs in vitro (Fig. 1b, c). qRT-PCR was performed to analyze the expression of LncRNA H19 among the MSC, pcDNA-H19-MSC, and si-H19-MSC groups. As shown in Fig. 1d, the expression of LncRNA H19 in MSCs was significantly upregulated by transfection of pcDNA-H19 but was inhibited after si-H19 treatment.

We then established a SAP model by retrograde injection of NaT and transplanted MSCs with various treatments into SAP rats via the tail vein as previously described [23]. To explore the effect of LncRNA H19 in MSCs used to treat SAP, we investigated the expression of LncRNA H19 in pancreatic tissues from different treatment groups. The results suggested that MSCs could enhance the expression of LncRNA H19, which was suppressed in the SAP group; meanwhile, the expression of LncRNA H19 in the pcDNA-H19-MSC group was much higher than that in the MSC group, but the expression of LncRNA H19 in the SAP and si-H19-MSC groups was not significantly different (Fig. 1e). Hematoxylin and eosin (H&E) staining of pancreatic tissues showed that transplantation of pcDNA-H19-MSCs could decrease pathological scores, which were enhanced in the SAP group (Fig. 1f, g). In addition, compared with the SAP and MSC groups, pcDNA-H19-MSCs clearly suppressed the serum levels of amylase, as well as serum lactate dehydrogenase (LDH), pancreatic LDH, and pancreatic pro-inflammatory mediators (IL-1β, IL-6, IL-8, and TNF-α); in contrast, si-H19-MSCs significantly reversed these protective effects in SAP (Fig. 1h–n). Therefore, LncRNA H19 increased the therapeutic efficacy of MSCs on SAP.

### LncRNA H19 suppressed autophagy in SAP

**Fig. 2:**
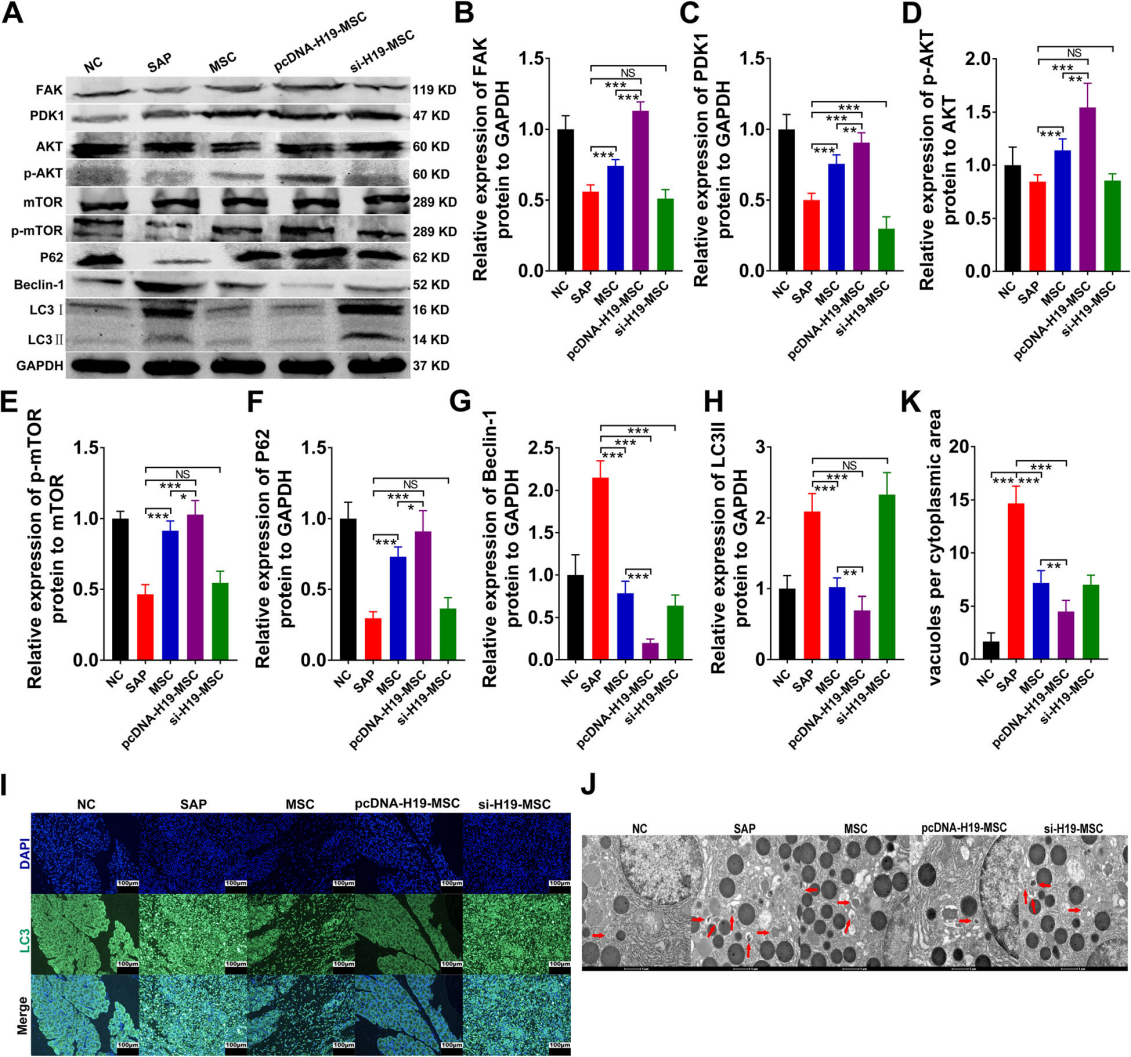
LncRNA H19 repressed autophagy in SAP. **a**–**h** Western blot analysis of FAK, PDK1, AKT, p-AKT, mTOR, p-mTOR, P62, Beclin-1, and LC3 protein levels in the pancreas. **i** Immunofluorescence staining of LC3 in the pancreas (scale bar = 100 μm). **j** Characteristic pictures of autophagosomes observed using TEM in the pancreas (scale bar = 1 μm). Red arrows show autophagosomes. **k** The percentage of autophagic vacuoles per cytoplasmic area. Data are demonstrated means ± SD from at least three separate experiments. *n* = 6/group. *p < 0.05, **p < 0.01, ***p < 0.001. NS, not significant

Western blot analyses were performed to determine protein expression of the FAK/PDK1/AKT/mTOR pathway, due to its crucial roles in the process of autophagy, which is associated with the development of SAP [25]. The western blot results showed that pcDNA-H19-MSCs enhanced the expression of FAK, PDK1, p-AKT, p-mTOR, and P62 but inhibited the expression of Beclin-1 and LC3 II, compared with the SAP and MSC groups (Fig. 2a–h). Simultaneously, there was very little difference between the si-H19-MSC and SAP groups in the protein levels of the FAK/PDK1/AKT/mTOR pathway (Fig. 2a–h). The findings from immunohistochemistry and immunofluorescence assays also supported the western blot results (Additional file 2 and Fig. 2i). Additionally, transmission electron microscopy was utilized to observe the ultrastructure of cells in pancreatic tissues, and transplanted pcDNA-H19-MSCs significantly reduced the number of autophagosomes (Fig. 2j, k). Thus, LncRNA H19 inhibited autophagy via activation of the FAK/PDK1/AKT/mTOR pathway in rats with SAP.

### LncRNA H19 facilitated proliferation in SAP

**Fig. 3:**
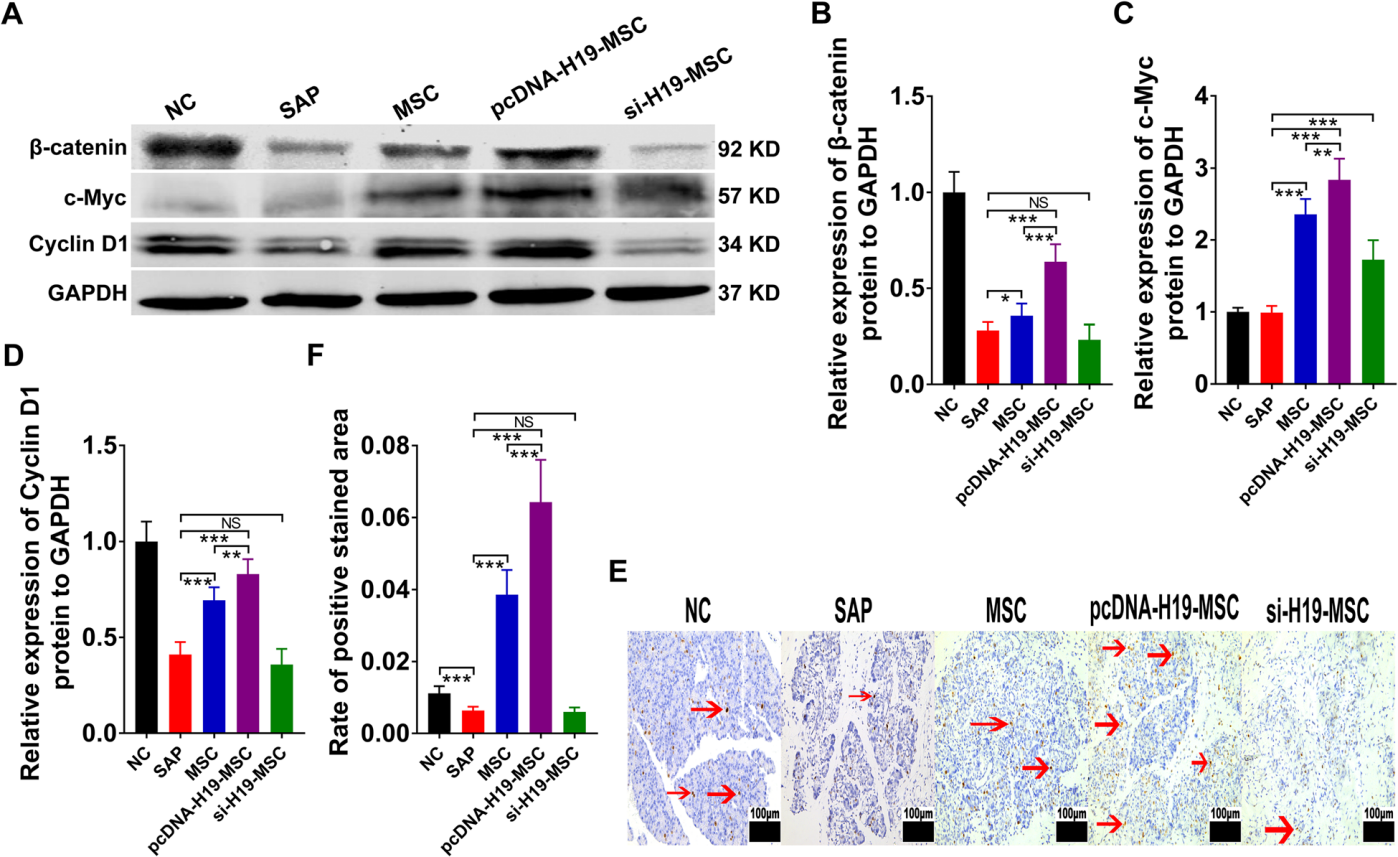
LncRNA H19 facilitate proliferation in SAP. **a**–**d** Western blot analysis of pancreatic β-catenin, c-Myc, and cyclin D1 protein levels. **e** Immunohistochemistry of Ki67 staining in the pancreas (scale bar = 100 μm) (red arrowhead indicates Ki67 staining-positive cells). **f** Proportion of Ki67-positive stained area. Data shown are means ± SD from at least three separate experiments. *n* = 6/group. *p < 0.05, **p < 0.01, ***p < 0.001. NS, not significant

β-Catenin belongs to the adhesion molecule family, and WNT/β-catenin signaling has been shown to promote cell proliferation in human melanomas [31]. To investigate the effect of LncRNA H19 on proliferation, we performed western blots of β-catenin and its targeted genes including c-Myc and cyclin D1. The results suggested that the expression of β-catenin, c-Myc, and cyclin D1 in the LncRNA H19 group was much higher than that in the SAP and MSC groups, while si-H19 MSCs reversed the overexpression of cell proliferation-related proteins (Fig. 3a–d). The results from immunohistochemistry assays also confirmed the western blot results (Additional file 3). Moreover, Ki67 staining analysis suggested that LncRNA H19 facilitated proliferation in SAP (Fig. 3e, f). Therefore, we conclude that LncRNA H19 may benefit the treatment of SAP by increasing cell proliferation.

### LncRNA H19 acted as a sponge for miR-138-5p, and inhibition of miR-138 restrained autophagy in SAP

**Fig. 4:**
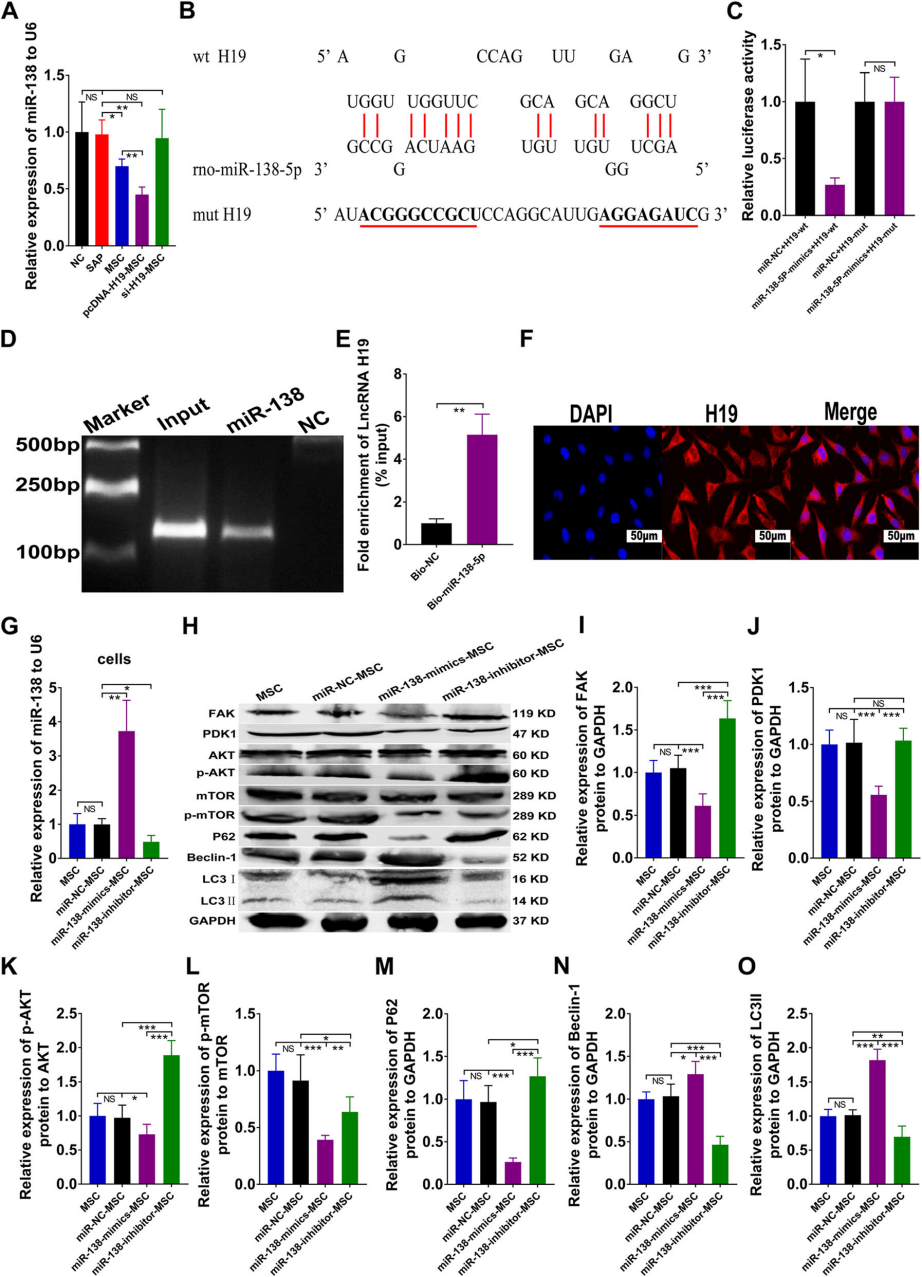
LncRNA H19 directly bonded with miR-138-5p and repression of miR-138 inhibited autophagy in SAP. **a** Relative mRNA expression of miR-138 in pancreatic tissues estimated by qRT-PCR. **b** Putative complementary sites within LncRNA H19 and rno-miR-138-5p were predicted by the Target Scan software. **c** Co-transfection of the 293T cells with LncRNA H19 and miR-138-5p mimics decreased the luciferase activity. **d** Agarose gel electrophoresis assay for the complex containing LncRNA H19 and biotin-coupled miR-138-5p or biotin-coupled miR-NC. **e** Fold enrichment of LncRNA H19 in capture assays. **f** Observation of localization of LncRNA H19 in the cytoplasm of MSCs by FISH assay, and the nuclei were stained blue, and LncRNA H19 was stained red (scale bar = 50 μm). **g** Relative mRNA expression of miR-138 in MSCs with different treatments analyzed by qRT-PCR. **h**–**o** Western blot analysis of pancreatic FAK, PDK1, AKT, p-AKT, mTOR, p-mTOR, P62, Beclin-1, and LC3 protein levels. Data represent means ± SD from at least three independent experiments. *n* = 6/group. *p < 0.05, **p < 0.01, ***p < 0.001. NS, not significant

We previously detected the expression of pancreatic miRNAs using qRT-PCR and discovered that the pcDNA-H19-MSC group had the lowest expression of miR-138 among the five groups (Fig. 4a). We determined that LncRNA H19 and rno-miR-138-5p had complementary base sequences by using the bioinformatics analysis software (Fig. 4b). Luciferase analysis was performed by inserting either the wild-type (wt) LncRNA H19 sequence or a mutant-type (mut) sequence into a luciferase construct, and the results indicated that luciferase activity was decreased after co-transfection of LncRNA H19-wt plasmid and miR-138-5p mimics into 293T cells as compared with the negative control (miR-NC; Fig. 4b, c). However, the activity of the mutant reporter did not show a significant difference after co-transfection with LncRNA H19-mut plasmid and miR-138-5p mimics as compared with miR-NC (Fig. 4b, c). Furthermore, less LncRNA H19 was acquired in the biotin-coupled miR-NC group than in the biotin-coupled miR-138-5p group (Fig. 4d, e), suggesting that LncRNA H19 could bind to miR-138-5p. In addition, fluorescence in situ hybridization (FISH) results showed that LncRNA H19 was localized to the cytoplasm of MSCs (Fig. 4f). We then transfected MSCs with miR-NC, miR-138 mimics, or miR-138 inhibitor in vitro and discovered using qRT-PCR that the MSCs transfected with miR-138 mimics expressed the highest level of miR-138 among the MSCs (Fig. 4g). Next, we transplanted these transfected MSCs into SAP rats 12 h after NaT induction. The western blot results indicated that miR-138-inhibitor-MSCs significantly upregulated the FAK/PDK1/AKT/mTOR pathway to suppress autophagy in SAP rats, but miR-138-mimic-MSCs exhibited the opposite result (Fig. 4h–o). Therefore, LncRNA H19 could adsorb miR-138-5p as a sponge RNA, and downregulating miR-138 could inhibit autophagy in SAP.

### LncRNA H19 directly bound miR-141-3p and repression of miR-141 boosted proliferation in SAP

**Fig. 5:**
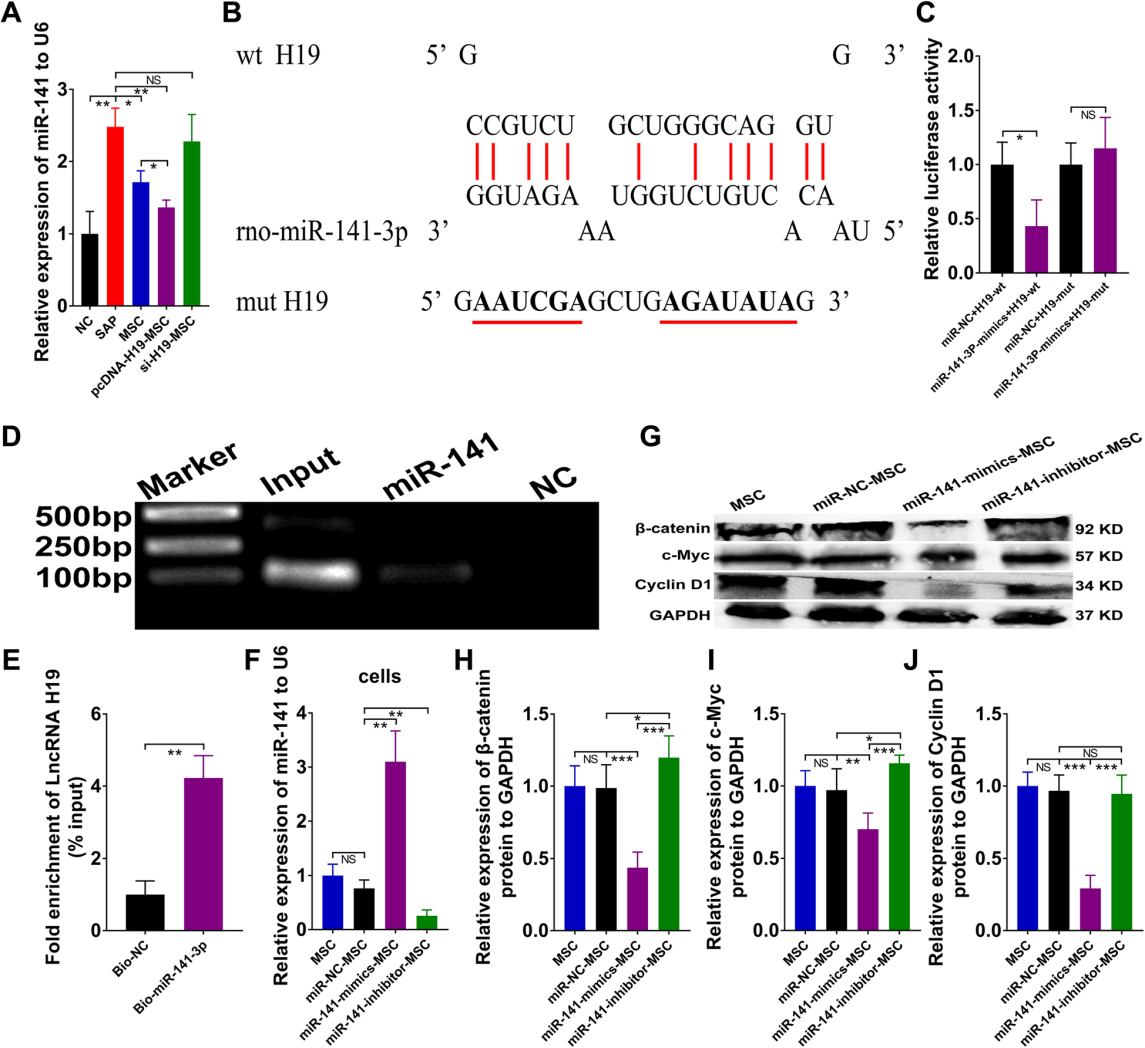
LncRNA H19 functioned as a sponge for miR-141-3p and suppression of miR-141 promote cell proliferation in SAP. **a** Relative mRNA expression of pancreatic miR-141 analyzed by qRT-PCR. **b** The schematic diagram illustrated the binding sites of LncRNA H19 with rno-miR-141-3p. **c** Co-transfection of the 293T cells with LncRNA H19 and miR-141-3p mimics reduced the luciferase activity. **d** Agarose gel electrophoresis assay for the complex containing LncRNA H19 and biotin-coupled miR-141-3p or biotin-coupled miR-NC. **e** Fold enrichment of LncRNA H19 in capture assays. **f** Relative mRNA expression of miR-141 in MSCs with various administrations detected by qRT-PCR. **g**–**j** Western blot analysis of pancreatic β-catenin, c-Myc, and cyclin D1 protein levels. Data shown are means ± SD from at least three independent experiments. *n* = 6/group. *p < 0.05, **p < 0.01, ***p < 0.001. NS, not significant

We previously examined the expression of miRNAs in pancreatic tissues and found that the expression of miR-141 in the pcDNA-H19-MSC group was the lowest (Fig. 5a). To investigate how LncRNA H19 mediates MSC treatment of SAP, miRNAs interacting with LncRNA H19 were identified using Target Scan software, and rno-miR-141-3p was predicted to be a potential target of LncRNA H19 (Fig. 5b). To further explore whether LncRNA H19 directly regulates miR-141-3p, we performed luciferase reporter assays. miR-NC or miR-141-3p mimics and LncRNA H19-wt or LncRNA H19-mut were co-transfected into 293T cells. Luciferase activity of the LncRNA H19-wt reporter was significantly decreased by miR-141 mimics, but luciferase activity of LncRNA H19-mut did not show any change after transfection with miR-141 mimics or miR-NC (Fig. 5c). In addition, more LncRNA H19 was captured in the biotin-coupled miR-138-5p group than in the miR-NC group (Fig. 5d, e), indicating direct binding of LncRNA H19 to miR-141-3p. The expression of miR-141 in MSCs was visibly increased after the transfection of miR-141 mimics, whereas miR-141 inhibitor transfection markedly reduced the expression of miR-141 (Fig. 5f). We found that suppression of miR-141 in MSCs significantly enhanced the expression of β-catenin and its targeted gene c-Myc; in contrast, miR-141-mimic-MSCs markedly suppressed the expression of β-catenin, c-Myc, and cyclin D1 (Figs. 5g–j). Thus, LncRNA H19 could act as an miRNA sponge for miR-141, and suppression of miR-141 promoted cell proliferation in SAP.

### PTK2 was a direct target of miR-138-5p, and overexpressing PTK2 repressed autophagy in SAP

**Fig. 6:**
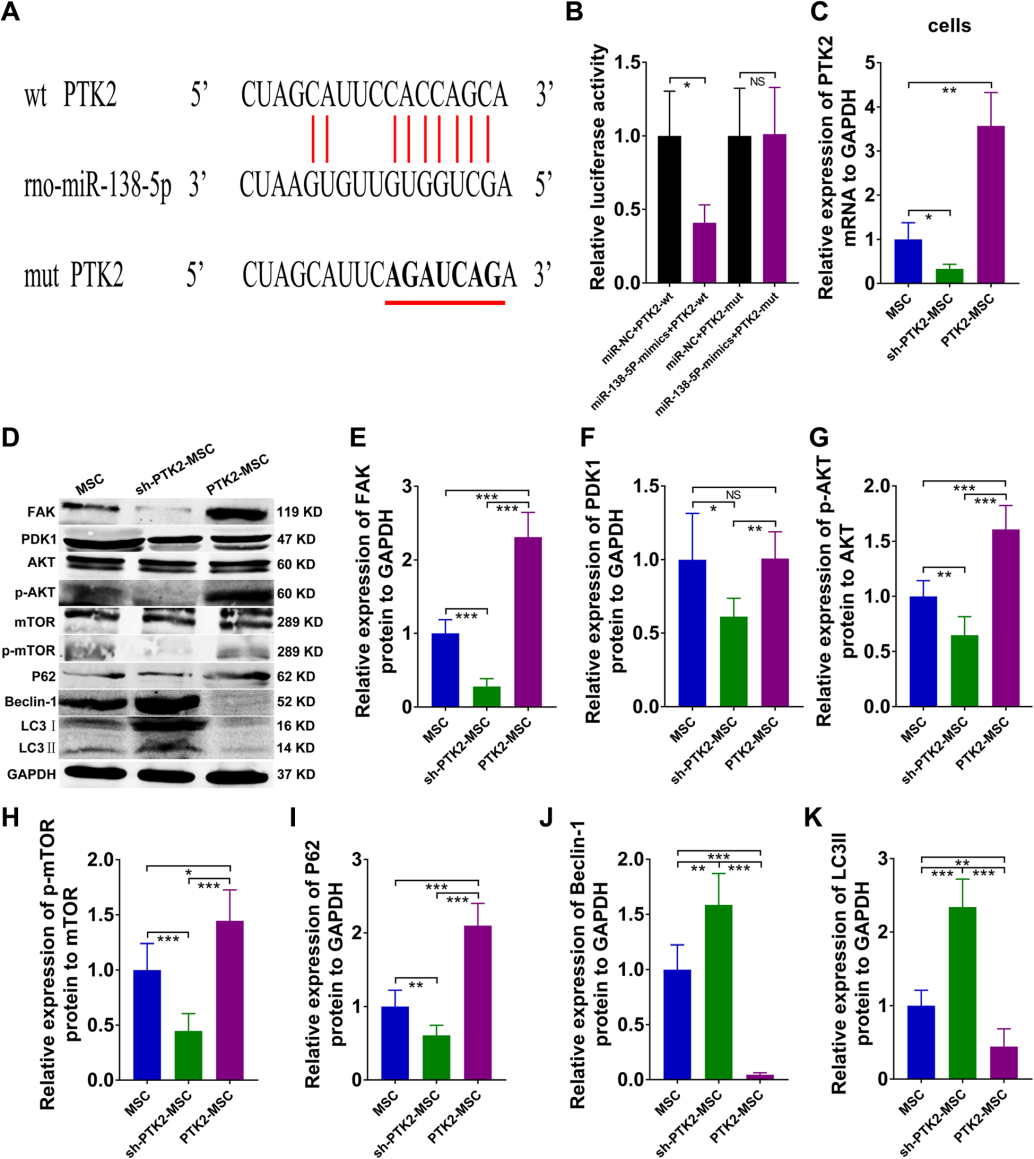
PTK2 targeted miR-138-5p directly, and upregulation of PTK2 repressed autophagy in SAP. **a** Potential bind sites of rno-miR-138-5p in PTK2 3′-UTR. **b** PTK2-wt or PTK2-mut plasmid was co-transfected with miR-NC or miR-138-5p mimics into 293T cells for 24 h, then luciferase activity was detected. **c** Relative mRNA expression of PTK2 in MSCs with different administrations analyzed by qRT-PCR. **d**–**k** Western blot analysis of pancreatic FAK, PDK1, AKT, p-AKT, mTOR, p-mTOR, P62, Beclin-1, and LC3 protein levels. Data represent means ± SD from at least three separate experiments. *n* = 6/group. *p < 0.05, **p < 0.01, ***p < 0.001. NS, not significant

Feng et al. have confirmed that protein tyrosine kinase 2 (PTK2), encoding focal adhesion kinase (FAK), acts as a candidate synthetic lethal gene [32]. In the present study, we have demonstrated that FAK plays an important role in regulating autophagy in MSCs used to treat SAP. miRNA-138 has been shown to control the osteogenic differentiation of human mesenchymal stem cells by targeting PTK2 directly [33], but few studies have focused on rat cells. We predicted the complementary sequences of PTK2 and rno-miR-138-5p using TargetScan (Fig. 6a). By performing luciferase reporter assays, we discovered that transfection of rno-miR-138-5p mimics could downregulate PTK2-wt, but not PTK2-mut (Fig. 6b). In vitro, short hairpin RNA (shRNA) of PTK2 (sh-PTK2) was constructed using the pGPU6 plasmid (GenePharma) to knock down PTK2, and a PTK2 overexpression plasmid was constructed with pEX2 plasmids (GenePharma; Fig. 6c). Western blot analysis suggested that upregulating PTK2 in MSCs could markedly enhance signaling in the FAK/PDK1/AKT/mTOR pathway to restrain autophagy in SAP, whereas downregulation of PTK2 successfully reversed this suppressive effect (Fig. 6d–k). Thus, miR-138-5p binds to PTK2 directly, and overexpressing PTK2 can repress autophagy in SAP.

### MiR-141-3p targeted β-catenin directly, and upregulation of β-catenin promoted proliferation in SAP

**Fig. 7:**
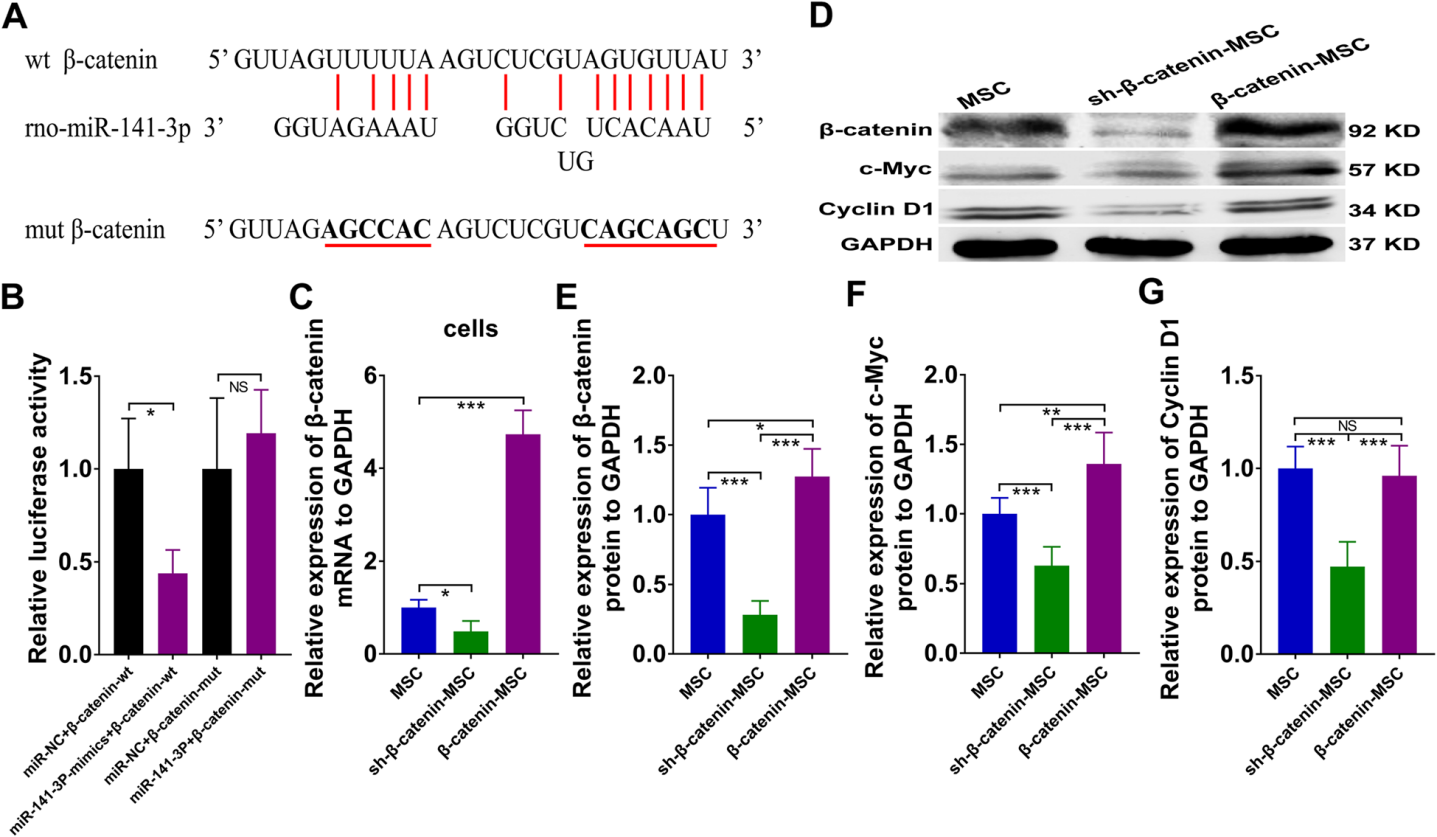
β-Catenin was a direct target of miR-141-3p, and overexpressing β-catenin boosted proliferation in SAP. **a** Feasible bind sites of rno-miR-141-5p in β-catenin 3′-UTR. **b** β-Catenin-wt or β-catenin-mut plasmid was co-transfected with miR-NC or miR-141-3p mimics into 293T cells for 24 h, then luciferase activity was examined. **c** Relative mRNA expression of β-catenin in MSCs with various treatments detected by qRT-PCR. **d**–**g** Western blot analysis of pancreatic β-catenin, c-Myc, and cyclin D1protein levels. Data represent means ± SD from at least three separate experiments. *n* = 6/group. *p < 0.05, **p < 0.01, ***p < 0.001. NS, not significant

We predicted the complementary sequences of rno-miR-141-3p and β-catenin using TargetScan (Fig. 7a). Luciferase assays suggested that co-transfection of miR-141-3p mimics with a β-catenin 3′-untranslated region (3′-UTR)-wt plasmid inhibited luciferase activity markedly compared to transfection of miR-NC or β-catenin 3′-UTR-mut (Fig. 7b). The expression of β-catenin in MSCs was upregulated by the transfection of a β-catenin overexpression plasmid; in contrast, the expression was downregulated by sh-β-catenin plasmid transfection (Fig. 7c). We discovered that overexpressing β-catenin in MSCs clearly upregulated the expression of β-catenin and c-Myc, whereas repression of β-catenin significantly suppressed the proliferation-related proteins (Fig. 7d–g). Therefore, β-catenin was a direct target of miR-141-3p, and the upregulation of β-catenin increased cell proliferation in SAP.

## Discussion

The incidence rate of AP in the USA and Europe is increasing about 5% per year, and approximately 20% of these AP cases will progress to SAP (necrotizing pancreatitis), which is characterized by 10–30% mortality, mainly due to infection of pancreatic necrotic tissue and infectious complications [34]. Hence, patients with SAP require multidisciplinary treatment from a team of gastroenterologists, interventional radiologists, and surgeons. Nevertheless, SAP mortality increases yearly [35]. In view of the current SAP therapeutic strategies, it is imperative to explore more effective treatment. Our group has demonstrated that MSC transplantation can successfully alleviate SAP by suppressing autophagy [25], but the underlying mechanisms remain unknown. We unexpectedly discovered that the expression of LncRNA H19 of pancreatic tissues was much higher in the MSC group than in the SAP group, but there have been no studies of the role of LncRNA H19 in SAP. Therefore, this important finding motivated us to further explore the underlying mechanisms by which LncRNA H19 regulated the therapeutic efficacy of MSCs in rats with SAP.

The levels of serum amylase are a typical biological marker of SAP [36], as is LDH [37]. SAP is associated with the involvement of abundant pro-inflammatory mediators, including IL-1β, IL-6, IL-8, and TNF-α [38]. As shown in Fig. 1, pcDNA-H19-MSCs exhibited a stronger anti-inflammatory effect than did MSCs, while downregulation of LncRNA H19 in MSCs markedly weakened their inhibition of systemic inflammatory responses. Thus, the results suggested that LncRNA H19 effectively increased the anti-inflammatory efficacy of MSCs on SAP.

As mentioned earlier, we previously confirmed that MSCs could repress autophagy to attenuate SAP by promoting the PI3K/AKT/mTOR signaling pathway [25]. LncRNA H19 has been shown to induce autophagy activation via the H19/SAHH/DNMT3B axis [26]. Meanwhile, LncRNA H19 overexpression suppressed autophagy of vascular smooth muscle cells in atherosclerosis [39]. These two examples illustrate that LncRNA H19 may regulate autophagy bidirectionally. Our results using a variety of methods demonstrated that the upregulation of LncRNA H19 in MSCs significantly restrained activated autophagy in rats with SAP by promoting the FAK/PDK1/AKT/mTOR signaling pathway, compared with the MSC and si-H19-MSC groups (Fig. 2).

β-Catenin, a type of transmembrane protein, is likely to play a crucial role in the progression and severity of AP [12]. Our previous research has determined that MSCs enhance the expression of β-catenin in SAP [20]. In the current study, the expression of the β-catenin in the pcDNA-H19-MSC group was much higher than that in the MSC and si-H19-MSC groups, as well as c-Myc and cyclin D1 (Fig. 3). The Wnt/β-catenin signaling pathway has been confirmed to increase cell proliferation and differentiation [40]. Thus, it is likely that overexpressed LncRNA H19 can promote the proliferation of pancreatic acinar cells in SAP via this pathway.

The function of lncRNAs is closely correlated with their localization within the cell [41]. LncRNAs can function as competing endogenous RNA when they are mainly located in the cytoplasm [42, 43]. We found that LncRNA H19 was mainly localized to the cytoplasm of MSCs using RNA FISH assays (Fig. 4). Then, to investigate the mechanism of LncRNA H19 in SAP, we utilized bioinformatics prediction to seek some miRNAs that could interact with LncRNA H19. Our research illustrated that LncRNA H19 could directly bind to rno-miR-138-5p and rno-miR-141-3p (Figs. 4 and 5). There have been no previous investigations to our knowledge of miR-138 in AP or SAP, but the expression of serum miR-141 was upregulated in AP [44]. Here, the suppression of miR-138-5p in MSCs significantly promoted the FAK/PDK1/AKT/mTOR pathway to repress autophagy in SAP (Fig. 4). In addition, knockdown of miR-141-3p in MSCs increased proliferation by upregulating the expression of β-catenin, c-Myc, and cyclin D1(Fig. 5).

Furthermore, we explored the potential target genes of rno-miR-138-5p and rno-miR-141-3p using TargetScan. Our results indicated that miR-138-5p and miR-141-3p directly targeted PTK2 (Fig. 6) and β-catenin (Fig. 7), respectively, and downregulated their expression. PTK2, encoding FAK, has been confirmed to mediate protective autophagy in anoikis-resistant glioma stem cells [45]. In our study, the upregulation of PTK2 in MSCs restrained autophagy via activation of the FAK/PDK1/AKT/mTOR signaling pathway in SAP (Fig. 6). Overexpression of β-catenin in MSCs effectively facilitated cell proliferation in SAP, along with the enhanced expression of c-Myc (Fig. 7).

There are still some weaknesses in our study, which we do not believe compromise our conclusions. We neglected the potential and meaningful consequences that concentration gradients and time may bring out, and we also ignored the possible side effects of cell therapy which may occur in the future. We will address these issues in future studies.

In summary, negative control rats treated with NaT developed SAP, along with activated autophagy and suppressed cell proliferation. As a competing endogenous RNA, LncRNA H19 sponges rno-miR-138-5p and rno-miR-141-3p enhance the expression of PTK2 and β-catenin and upregulate the FAK/PDK1/AKT/mTOR signaling pathway and β-catenin target genes (c-Myc and cyclin D1), while inhibiting autophagy and promoting proliferation. Hence, LncRNA H19 may provide us with a novel strategy utilizing MSC therapy for SAP in the future.

## Conclusion

When the pancreas is critically damaged, AP or even SAP may injure the organism, causing excessive autophagy and suppressed cellular proliferation. MSCs can be utilized to attenuate SAP, and LncRNA H19 markedly enhances their therapeutic efficacy. During MSC treatment, upregulated LncRNA H19 can act as an miRNA sponge to adsorb rno-miR-138-5p and rno-miR-141-3p, facilitating the expression of PTK2 and β-catenin, which in turn increases FAK/PDK1/AKT/mTOR signaling to suppress autophagy and promotes cell proliferation (Additional file 4).

## Supplementary information

**Additional file 1.** A supplementary image with the experimental groups.

**Additional file 2.** Findings from immunohistochemistry and immunofluorescence assays.

**Additional file 3.** Results from immunohistochemistry assays.

**Additional file 4.** During MSC treatment, upregulated LncRNA H19 can act as an miRNA sponge to adsorb rno-miR-138-5p and rno-miR-141-3p, facilitating the expression of PTK2 and β-catenin, which in turn increases FAK/PDK1/AKT/mTOR signaling to suppress autophagy and promotes cell proliferation.

## Data Availability

The datasets used and/or analyzed during the current study are available from the corresponding authors on reasonable request.

## Abbreviations

SAP: Severe acute pancreatitis
MSCs: Mesenchymal stem cells
LncRNA H19: Long noncoding RNA H19
NaT: Sodium taurocholate
FAK: Focal adhesion kinase
PTK2: Protein tyrosine kinase 2
AP: Acute pancreatitis
IL: Interleukin
TNF-α: Tumor necrosis factor alpha
siRNA: Small interfering RNA
miR-NC: miRNA negative control
shRNA: Short hairpin RNAs
LDH: Lactate dehydrogenase
PDK1: Phosphoinositide-dependent kinase 1
AKT: Protein kinase B
p-AKT: Phosphorylated AKT
mTOR: Mammalian target of rapamycin
p-mTOR: Phosphorylated mTOR
LC3: Light chain 3
DAPI: 4,6-Diamidino-2-phenylindole
FITC: Fluorescein isothiocyanate
H&E: Hematoxylin and eosin
