# Supplementary figures and images for: Long noncoding RNA H19 regulates the therapeutic efficacy of mesenchymal stem cells in rats with severe acute pancreatitis by sponging miR-138-5p and miR-141-3p
Source: Stem Cell Res Ther. 2020 Sep 25;11:420. doi: 10.1186/s13287-020-01940-z (PMC7519546; doi:10.1186/s13287-020-01940-z)

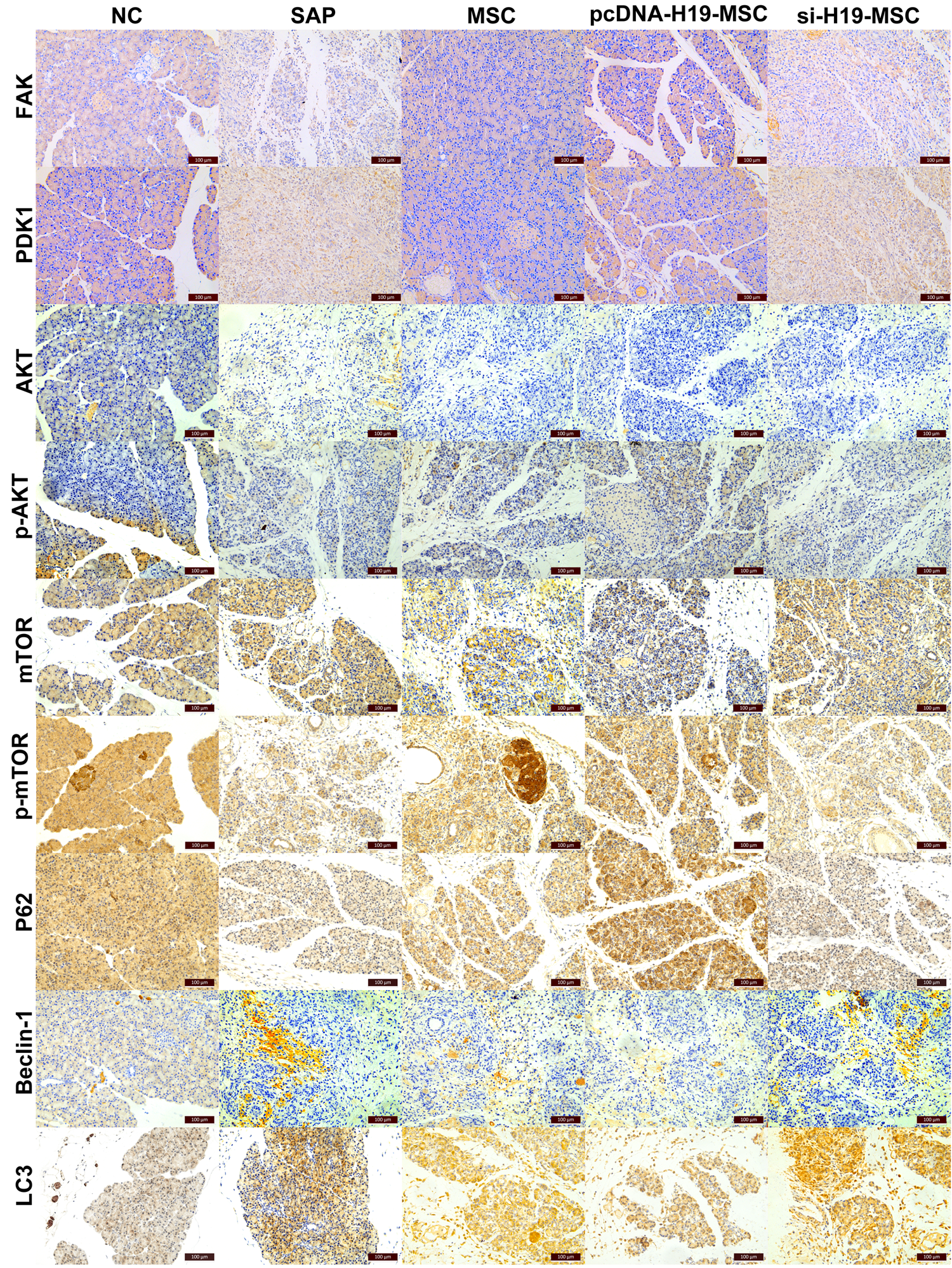

Supplement: Supplementary file 2 — Additional file 2. Findings from immunohistochemistry and immunofluorescence assays. [file 13287_2020_1940_MOESM2_ESM.tif]

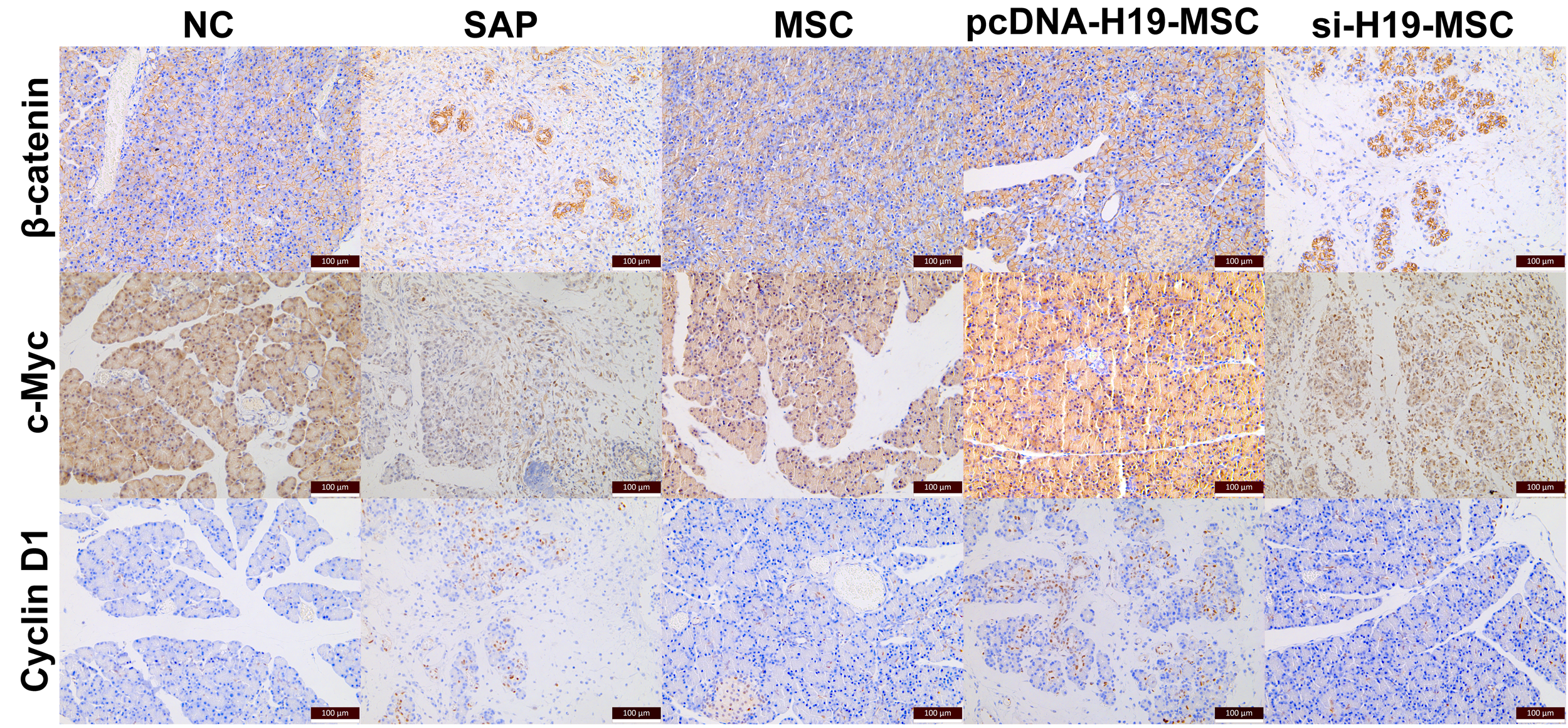

Supplement: Supplementary file 3 — Additional file 3. Results from immunohistochemistry assays. [file 13287_2020_1940_MOESM3_ESM.tif]

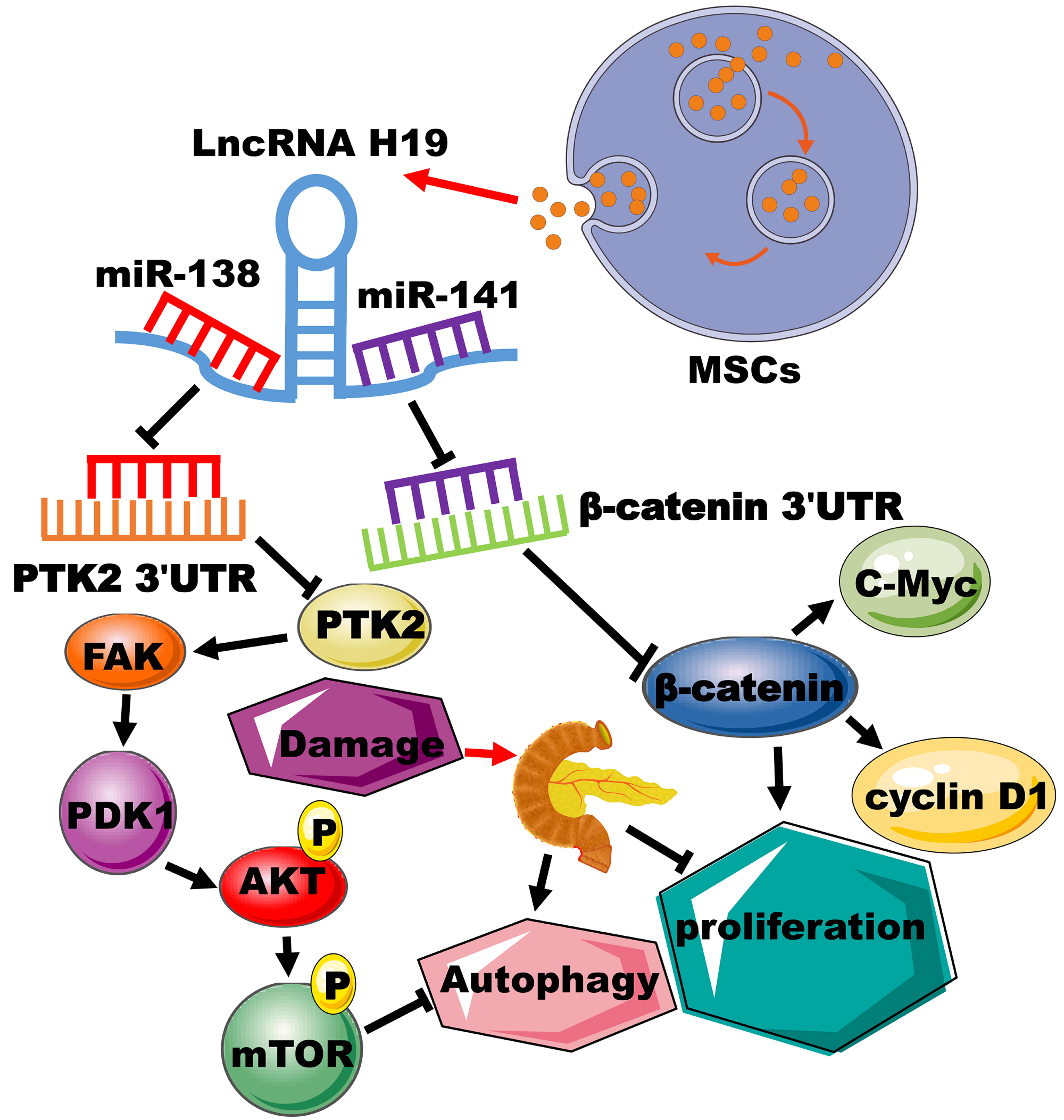

Supplement: Supplementary file 4 — Additional file 4. During MSC treatment, upregulated LncRNA H19 can act as an miRNA sponge to adsorb rno-miR-138-5p and rno-miR-141-3p, facilitating the expression of PTK2 and β-catenin, which in turn increases FAK/PDK1/AKT/mTOR signaling to suppress autophagy and promotes cell proliferation. [file 13287_2020_1940_MOESM4_ESM.tif]
